# The MID1 Protein: A Promising Therapeutic Target in Huntington’s Disease

**DOI:** 10.3389/fgene.2021.761714

**Published:** 2021-10-01

**Authors:** Annika Heinz, Judith Schilling, Willeke van Roon-Mom, Sybille Krauß

**Affiliations:** ^1^ University of Siegen, Institute of Biology, Human Biology / Neurobiology, Siegen, Germany; ^2^ German Center for Neurodegenerative Diseases (DZNE), Bonn, Germany; ^3^ Leiden University Medical Center, Department of Human Genetics, Leiden, Netherlands

**Keywords:** MID1, Huntington’s disease, Alzheimer’s disease, mRNA translation, RNA-protein interaction, CAG repeat, RNA-targeting drug

## Abstract

Huntington’s disease (HD) is caused by an expansion mutation of a CAG repeat in exon 1 of the huntingtin (*HTT*) gene, that encodes an expanded polyglutamine tract in the HTT protein. HD is characterized by progressive psychiatric and cognitive symptoms associated with a progressive movement disorder. HTT is ubiquitously expressed, but the pathological changes caused by the mutation are most prominent in the central nervous system. Since the mutation was discovered, research has mainly focused on the mutant HTT protein. But what if the polyglutamine protein is not the only cause of the neurotoxicity? Recent studies show that the mutant RNA transcript is also involved in cellular dysfunction. Here we discuss the abnormal interaction of the mutant HTT transcript with a protein complex containing the MID1 protein. MID1 aberrantly binds to CAG repeats and this binding increases with CAG repeat length. Since MID1 is a translation regulator, association of the MID1 complex stimulates translation of mutant HTT mRNA, resulting in an overproduction of polyglutamine protein. Thus, blocking the interaction between MID1 and mutant HTT mRNA is a promising therapeutic approach. Additionally, we show that MID1 expression in the brain of both HD patients and HD mice is aberrantly increased. This finding further supports the concept of blocking the interaction between MID1 and mutant HTT mRNA to counteract mutant HTT translation as a valuable therapeutic strategy. In line, recent studies in which either compounds affecting the assembly of the MID1 complex or molecules targeting HTT RNA, show promising results.

## Introduction

HD is a monogenic disorder of the central nervous system that was first described in 1872 by George Huntington (Huntington, 2003). HD is caused by an expansion mutation of a CAG repeat within the *HTT* gene. This CAG repeat translates into a polyglutamine tract in the HTT protein. Since the mutation was discovered in the 1990s (MACDONALD, 1993), research mainly focused on the mutant polyglutamine protein, which forms aggregates in the patient’s brain and is considered a pathological hallmark. However, in the last couple of years several studies showed that it is not solely the polyglutamine protein that drives pathogenic processes, but the mutant RNA transcript is also involved in cellular dysfunction [reviewed in (Nalavade et al., 2013; Schilling et al., 2016; Heinz et al., 2021)]. The main concept of a toxic gain-of-function of the expanded CAG repeat RNA is that the mutant CAG repeat represents an aberrant binding site for RNA-binding proteins, that get sequestered to and carry out an abnormal function in conjunction with the CAG repeat RNA. For example, the mutant HTT transcript aberrantly recruits the Midline1 (MID1) protein. MID1 first aroused attention in 1997, when mutations in MID1 were discovered as the cause of Opitz-BBB/G syndrome (OS), which is characterized by malformations of the ventral midline. These include hypospadias, hypertelorism, heart defects, cleft lip and palate and structural brain abnormalities. In addition, intellectual disability and/or developmental delay occur in approximately 30 percent of affected individuals. Since the discovery that MID1 is involved in the pathogenesis of OS, several functional studies on this protein were performed. MID1 belongs to the protein family of RING finger proteins and contains several conserved domains: a RING finger, two B-Boxes, a coiled-coil domain, a Fibronectin type-III domain, and a B30.2/SPRY domain. While all these domains generally are involved in diverse protein-protein or nucleic acid-protein interactions, some of the domains of MID1 have specific known functions: for example, *via* its RING finger domain, MID1 executes an E3 ubiquitin ligase activity, MID1 forms homo-dimers via its coiled-coil domain, and MID1 associates to microtubules *via* its C-terminal domains. To date several studies have been conducted that indicate that MID1 is a central player in the cell and is involved in several cellular processes, both in healthy tissue as well as in diseases [reviewed in (Winter et al., 2016)]. However, after the discovery that MID1 is responsible for OS, it took another decade until the first connection between MID1 and neurodegenerative diseases was found (Kickstein et al., 2010). With respect to neurodegenerative diseases, one important aspect of MID1 function is its ability to bind to mRNA and induce translation. This translation inducer function of MID1 can be explained by the recruitment of the translation machinery to the respective MID1 target mRNA. In this respect, MID1 has been shown to assemble a protein complex that contains various subunits of both the eukaryotic translation initiation factor complex (eIF complex) and the ribosome (Matthes et al., 2018a). This translational regulator function is of particular interest in neurodegenerative diseases and had first been linked to Alzheimer’s disease (AD). AD is the most common form of dementia in the elderly and is characterized by two neuropathological hallmarks: intracellular neurofibrillary tangles, that are mainly composed of an aberrantly high phosphorylated and thus aggregate-prone protein called Tau, and extracellular amyloid plaques, which are mainly formed by the Aβ peptide, which results from aberrant cleavage of the amyloid precursor protein (APP). Strikingly, MID1 interacts with mRNAs and proteins involved in formation of both of these pathological hallmarks. On one hand, by regulating the ubiquitin-dependent degradation of PP2A, the major Tau phosphatase, MID1 can influence Tau phosphorylation (Kickstein et al., 2010; Schweiger et al., 2017). On the other hand, by binding to and regulating the translation of two mRNAs that are involved in formation of Aβ (the APP mRNA and the BACE1 mRNA), MID1 also influences the extracellular amyloid plaques (Hettich et al., 2014; Matthes et al., 2018a). Analysis of the expression level of MID1 in AD brain tissue suggest that MID1 is aberrantly high expressed in patients and thus an abnormally high MID1 activity may push pathogenesis in the course of neurodegeneration (Schweiger et al., 2017). In 2013, a first connection between MID1 and polyglutamine disorders like HD had been shown (Krauss et al., 2013). Here, the mutant HTT transcript aberrantly recruits the MID1 protein complex and this binding increases with CAG repeat length. MID1 in turn recruits the translation machinery to the expanded CAG repeat mRNA, and thus, association of the MID1 complex drives an aberrantly high translation of mutant HTT mRNA, resulting in overproduction of mutant polyglutamine protein.

## MID1 in Huntington’s Disease

### MID1 and CAG Repeats

CAG repeat expansion diseases including HD represent one of the most common forms of inherited neurodegeneration. The expansion mutation of the trinucleotide CAG in the disease-causing transcripts leads to an aberrant folding of the RNA into a hairpin structure. These hairpins increase in length and stability with increasing CAG repeat numbers (Sobczak et al., 2003; Krzysztof and Wlodzimierz, 2005; Kiliszek et al., 2010; de Mezer et al., 2011) and aberrantly recruit various RNA binding proteins (RBP). This sequestration of RBPs may either lead to loss of normal function or induce aberrant function of these proteins in conjunction with the RNA.

**FIGURE 1:**
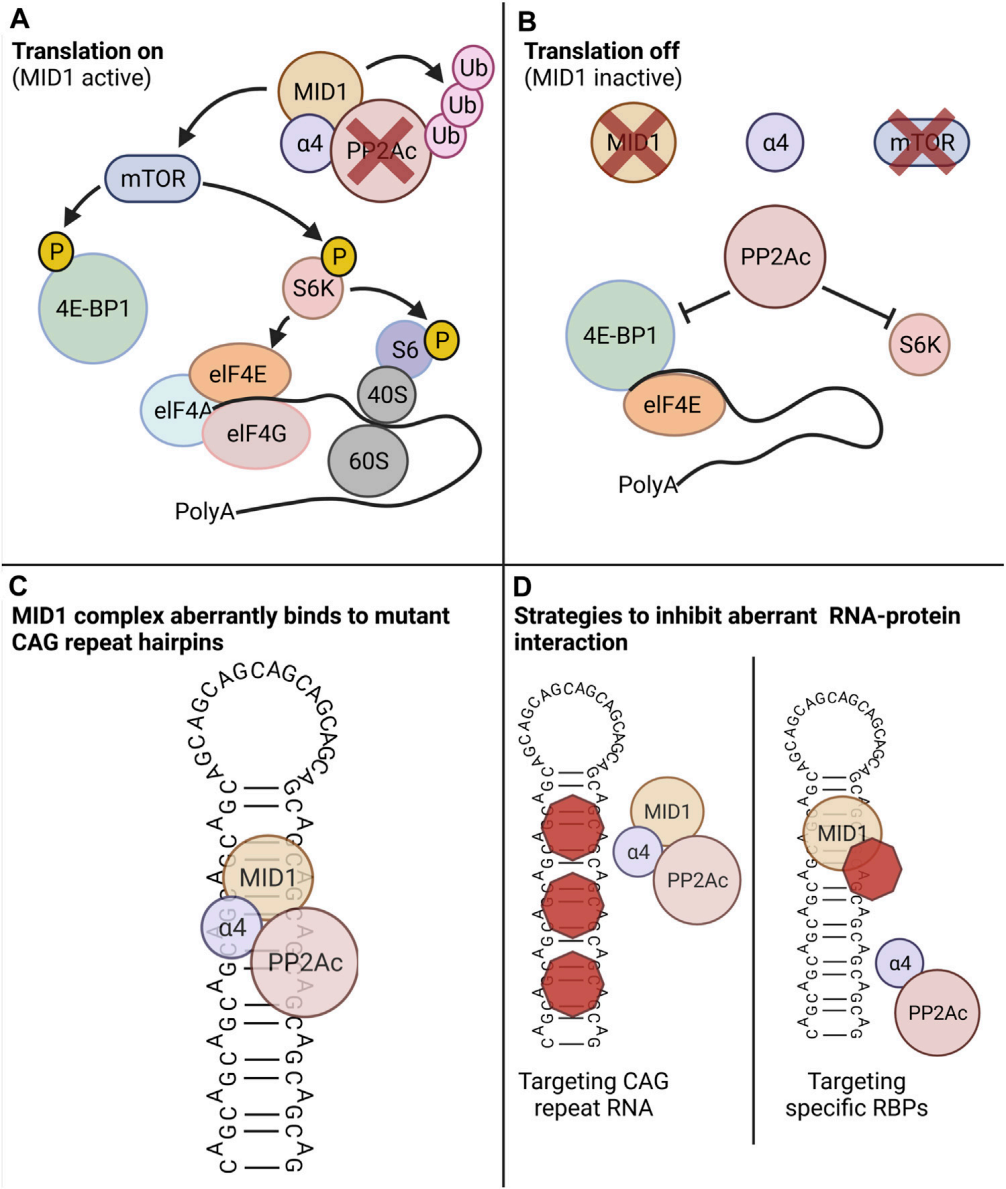
The MID1 protein complex regulates translation in a PP2A- and mTOR-dependent manner. PP2A and its counteractor mTOR control the phospho-dependent activity of the translation regulators S6K and 4E-BP1. **(A)** Scenario in which MID1 is active: MID1 ubiquitinates and thereby inactivates PP2A and at the same time it activates mTOR. 4E-BP1, a negative regulator of translation that suppresses translation when bound to the 5′ end of an RNA, gets phosphorylated by mTOR. This leads to the detachment of 4E-BP1 from RNA and the release of this translational block. At the same time mTOR phosphorylates and thereby activates S6K. S6K then phosphorylates its target S6, which is a subunit of the ribosome. These two phosphorylation events promote ribosome assembly and promote translation initiation. **(B)** Scenario in which MID1 is inactive: PP2A is activated and mTOR inactivated. Thus, S6K and 4E-BP1 are dephosphorylated resulting in translation inhibition. **(C)** Schematic illustration of three-dimensional CAG-repeat RNA hairpin, in which with the opposing strands of the CAG repeat forming Watson-Crick contacts between the G- and C-positions and wobble pairs at the A-A mismatches. MID1 recruits its interacting proteins including PP2A and S6K to the RNA hairpin and thereby indirectly stimulates translation. **(D)** Novel treatment strategies that can counteract the aberrant RNA-protein interaction of the MID1-complex and mutant CAG-repeat hairpin. Left: compounds that bind to the CAG-repeat hairpin can diminish the binding of the MID1-complex to the RNA. Left: compounds that disassemble the MID1 complex can diminish MID1-dependent aberrant translation of mutant CAG-repeat RNA. Created with BioRender.com.

The MID1 protein represents such an aberrant interaction partner. Its binding leads to an aberrant translation induction of mutant HTT mRNA. MID1 assembles a protein complex with its interaction partners PP2A and 40S ribosomal S6 kinase (S6K) and recruits this complex to the mutant HTT mRNA (Krauss et al., 2013). PP2A is composed of several subunits: the catalytic subunit PP2Ac binds to different regulatory subunits that mediate substrate specificity and subcellular localization. MID1 binds to PP2A *via* its regulatory subunit alpha4. Upon binding MID1 ubiquitinates and thereby targets PP2Ac for proteasomal degradation (Trockenbacher et al., 2001; Meroni and Diez-Roux, 2005). MID1 knockdown results in increased levels of PP2Ac, which in complex with its regulatory B-alpha subunit inhibits the binding of mTOR/Raptor and thus the activity of the mTOR/Raptor complex. Thus, MID1 regulates the composition of the mTOR/Raptor complex and thereby the activity of the kinase mTOR (mammalian target of rapamycin) in the mTORC1 protein complex (mTOR, mLST8 (mammalian lethal with SEC13 protein 8), raptor (regulatory-associated protein of mTOR) (Liu et al., 2011). Among other functions, mTOR phosphorylates proteins involved in translation regulation including S6K and 4E-BP1 (eukaryotic translation initiation factor 4E (eIF4E)-binding protein 1). This mTOR dependent phosphorylation activates S6K and inactivates the translation inhibitor 4E-BP1 (Nojima et al., 2003). Activated S6K phosphorylates and thereby activates a ribosomal subunit, S6. Phospho-inactivated 4E-BP1 is released from the 5′ end of the RNA and detaches from its interaction partners elF4E (eukaryotic translation initiation factor 4E). elF4E then binds to elF4G and elF4A, and the resulting complex binds to the 5′UTR followed by conformational changes of the RNA and the recruitment and assembly of the ribosome (Figure 1).

MID1 stimulates the translation rate of its target mRNAs by regulating the activities of PP2A and mTOR and thus the phosphorylation status of S6K and 4E-BP1. Thus, the CAG-repeat length-dependent binding of the MID1-protein complex leads to an increased translation rate of mutant HTT mRNAs, resulting in a toxic gain of function. Interestingly, this phenomenon of translational induction by MID1 is also found in models of other CAG repeat diseases, since the association of the MID1-complex to CAG repeats is independent of the repeat-flanking sequences (Griesche et al., 2016). Consequently, pharmacological targeting of the MID1 complex represents a promising approach to suppress the aberrant translation of expanded CAG repeat mRNA in HD and other CAG repeat diseases. However, one important aspect of a MID1-targeting strategy in HD is its expression in disease-tissue.

### MID1 Expression in HD

**FIGURE 2:**
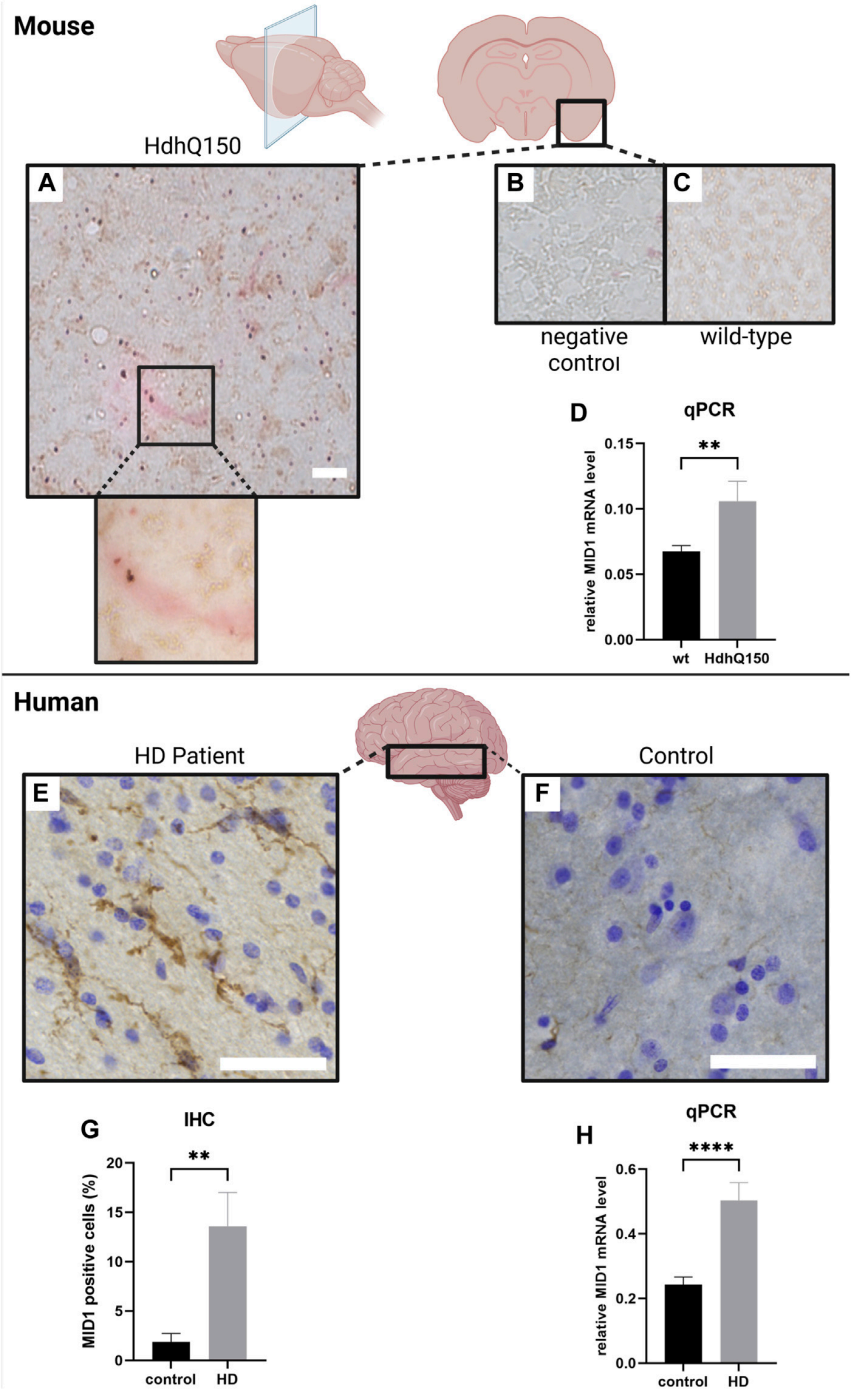
MID1 expression is increased in HD (mouse model (HdhQ150) and patients). MID1 expression in 10–12 week-old mice was analyzed by immunohistochemistry (IHC) an qPCR. **(A)** IHC staining in coronal cortical sections of transgenic (tg) HdhQ150 mice. Sections were immuno-stained for MID1 (red) and HTT (brown). **(B)** Negative control staining without primary antibodies. Scale bar 100 µm. **(C)** IHC staining of MID1 and HTT in wild-type (wt) control animals. **(D)** MID1 qPCR analysis of cortical tissue from wt and tg animals was performed. MID1 levels were normalized to GAPDH. Columns represent mean values+/-SE, *p*-values are the result of a Mann-Whitney test (n_wt_ = 10, n_tg_ = 9). MID1 expression was analyzed in cortical tissue of HD patients and controls. Sections from the middle temporal gyrus (MTG) were immuno-stained for MID1 and cresyl violet was used for Nissl staining. Typical examples of sections are shown for HD patients **(E)** and control brains **(F)**. Scale bar 100 µm. **(G)** Quantification of MID1 positive cells (%) in IHC analysis of HD patients and controls. n = 6, p**<0.005 **(H)** qPCR analysis of MID1 expression normalized to RPL22 in cortex of HD patients and controls. n_control_ = 8, n_HD_ = 9, p****<0.0001. Columns represent mean values+/-SE, *p*-values are the result of a Mann-Whitney test. Created with BioRender.com.

Previous studies showed that the MID1 protein is ubiquitously expressed at low or medium levels (https://www.proteinatlas.org/ENSG00000101871-MID1/tissue (0.6.08.21)). However, the expression pattern of MID1 in HD brain tissue remained obscure. To address this, we analyzed MID1 expression in an HD mouse model, HdhQ150 (B6.129P2-Htt < tm2Detl>150J). Interestingly, at the early age of 10–15 weeks (young adults compared to humans), a more excitable state characterized by a complex change in neuronal activity pattern and hyperactive neurons, which is accompanied by behavioral changes is observed in this mouse model. Thus, we decided to use 10–12 week-old mice for our analysis. We first isolated RNA from cortex of 10-week-old transgenic (tg) and wild-type (wt) animals. Quantitative real-time PCR (qPCR) analysis revealed a significant upregulation of MID1 in cortex HD animals. In line with this result we also detected significantly upregulated MID1 protein levels by immunohistochemistry. Staining of MID1 in coronal sections of these animals revealed that MID1 is broadly expressed throughout the cortex. It is interesting to note that MID1 tends to occur more frequently around HTT protein aggregates (Figure 2). Together our data show that MID1 expression is increased in cortex of an HD mouse model.

Next, we asked if such an increase in expression of MID1 is also detectable in human HD brains. To address this, we performed expression analysis of MID1 in post-mortem brain tissue of nine HD patients and nine healthy controls. QPCR analysis revealed a significant upregulation of MID1 in the middle temporal gyrus of HD patients. In line with this result, increased MID1 expression was also detected in immunohistochemistry analysis: the number of MID1-positive cells is significantly higher in HD patients (Figure 2). Interestingly, MID1 expression is increased on transcript and protein level, arguing for an induction on the transcriptional level rather than protein stabilization or upregulation of the translation rate of MID1. Future studies should aim at identifying the underlying mechanism that induces MID1 expression in HD. Known regulators of MID1 expression include for example Downstream regulatory element antagonist modulator (DREAM) (Dierssen et al., 2012), Tumour necrosis factor-related apoptosis-inducing ligand (TRAIL) (Collison et al., 2015), and microRNAs miR-19, miR-340, miR-374 and miR-542 (Unterbruner et al., 2018).

Taken together, our data show that MID1 expression is aberrantly increased in HD brains, both in patients as well as in a mouse model. This together with the aberrant activity of MID1 on mutant HTT protein synthesis makes MID1 a promising target of an intervention strategy.

### Targeting MID1

As described above, targeting of MID1 represents a promising approach to reduce mutant HTT protein expression. In line with this, different experimental approaches targeting the interaction between MID1 and mutant HTT mRNA gave promising results. Two different strategies to disturb the interaction between the MID1 protein complex and the mutant CAG repeat RNA have been followed thus far: either targeting the CAG repeat RNA, or targeting the assembly of the MID1 protein complex. Following the first strategy, furamidine was identified as CAG repeat RNA binder in an *in silico* approach. Binding of furamidine to the CAG repeat interferes with the attachment of MID1 to HTT RNA. By displacing the MID1 complex from the CAG repeat RNA, furamidine reduces HTT protein synthesis. Thus, small molecules masking the CAG repeat RNA and thereby suppress MID1-dependent translation, represent an interesting therapeutic option (Matthes et al., 2018b).

The second strategy to inhibit the MID1-dependent translation of mutant HTT is targeting the MID1 protein. The assembly of MID1 with its interacting proteins alpha4 and PP2Ac is crucial for its translation regulator function. Interestingly, the antidiabetic drug metformin activates PP2A activity by inhibiting the binding of PP2Ac to its negative regulators MID1 and alpha 4 (Kickstein et al., 2010). Thereby metformin diminishes aberrant HTT translation and also restores early functional changes and behavioural aberrations in an HD mouse model (Arnoux et al., 2018). In another study, a peptide that is able to disrupt the interaction between MID1 and alpha4 was used disrupt the MID1-complex within cells. As expected, this results in decreased HTT protein expression in cultures of neurones derived from HD mice (Monteiro et al., 2018). Together, these data validate that the MID1 complex is a valuable drug target and pave the way for the further development and refinement of small molecule inhibitors of this interaction as potential therapies for HD.

## Discussion and Outlook

Protein expression from specific mRNA molecules is tightly regulated in a cell-type-dependent manner. In disease conditions, aberrant mRNA-protein interactions can occur and modify translation of disease-associated mRNAs. One example of such an aberrant interaction is binding of the MID1 to mutant HTT mRNA (Krauss et al., 2013). Functionally, MID1 induces protein translation of its target mRNAs (Krauss et al., 2013; Hettich et al., 2014; Köhler et al., 2014; Griesche et al., 2016), and in HD cell models, the interaction of MID1 and mutant HTT RNA leads to an increased translation rate of neurotoxic polyglutamine protein (Krauss et al., 2013). Thus, MID1 may be a disease modifier in HD. However, the expression level as well as the expression pattern of MID1 in HD brain tissue has not been studied thus far and previous studies mainly described MID1 expression in embryos or very young individuals. In mice MID1 is expressed in different tissues depending on the age. In embryos between E8.5–14.5 as well as in newborn animals it is expressed in the central nervous system [reviewed in (Winter et al., 2016)]. In humans MID1 is also expressed in different tissues depending on age, in 5–7 weeks old embryos in the central nervous system [reviewed in (Winter et al., 2016)]. In line, our data show that MID1 is detectable in central nervous system. However, the tissues we tested here were from mature individuals (adult). Strikingly, our data show a significantly upregulated expression of MID1 under disease-conditions, supporting our hypothesis that MID1 by pushing towards polyglutamine protein synthesis is a disease-modifier in HD and targeting of MID1 may represent one therapeutic approach to halt HD disease progression. In line, different studies in which either the binding of MID1 to mutant HTT mRNA (Matthes et al., 2018b) or the assembly of the MID1 protein complex had been pharmacologically blocked (Arnoux et al., 2018), showed reduced HTT translation and reduced behavioral aberrations in HD models. Recent studies show that lowering HTT levels in HD (in antisense oligonucleotide (AON) therapies) is a promising therapeutic approach (Kordasiewicz et al., 2012; Østergaard et al., 2013; Sciences, 2021). However, HTT is an essential protein and thus, it is possible that permanent reduction in HTT expression may cause long-term side-effects. In line, recent clinical trials targeting both, normal and mutant alleles, give first evidence that lowering HTT below a critical level may not be tolerated well (Deeprose and Rosser, 2021; Heinz et al., 2021). On benefit of targeting MID1 to reduce HTT protein expression over targeting the HTT transcript itself is that MID1 mostly acts on the disease allele, while keeping expression of the normal allele untouched. Furthermore, MID1 is aberrantly upregulated in HD and should be normally expressed at a low level, thus, reducing MID1 in HD may just bring the expression back to normal and therefore may cause less side-effects. Moreover, loss of function of MID1 (like in Opitz syndrome) leads developmental problems, but causes no adult-life effects. Thus, targeting MID1 in adults has most likely no detrimental effect.

## Data Availability

The original contributions presented in the study are included in the article/Supplementary Material, further inquiries can be directed to the corresponding author.
