# Supplementary material for: The MID1 Protein: A Promising Therapeutic Target in Huntington’s Disease
Source: Front Genet. 2021 Oct 1;12:761714. doi: 10.3389/fgene.2021.761714 (PMC8517220; doi:10.3389/fgene.2021.761714)
Supplement: Supplementary file 1 [file DataSheet1.docx]

Supplementary Material

# Methods

## Mice

HdhQ150 (B6.129P2-Htt<tm2Detl>150J) mice were maintained in a temperature-controlled room (22°C, 60% air humidity) with a light/dark cycle of 12h/12h and had access to food and water ad libitum. Animals were sacrificed by cervical dislocation and brains were snap-frozen in liquid nitrogen and stored frozen at -80°C until further analysis. All procedures were in compliance with German Animal Protection Law and were approved by the state government of North Rhine-Westphalia, Germany (84-02.04.2014.A202).

## Human post-mortem brain samples

Human brain tissue was collected and stored as previously described (1). Tissue was obtained with the families’ full consent and with the approval of the Leiden University Medical Center Institutional Ethics Committee. Detailed information about patients is given in table 1.

## Immunohistochemistry

### Mice

Sections were cut using microtome from fixed-frozen WT and HdhQ150 brain in OCT compound. The sections were cut at either 10 – 20 µm and collected on Labsolute adhesion slides (Th.Geyer) at -80°C. In use they were thawed and dried for 15 min, fixed in methanol for 10 min and finally dried again for 10 min. The sections were rehydrated for 10 min with PBS-T (0.05% Tween). To stain MID1 (2) (1:50 in 1% BSA in PBS-T) and HTT (Merck; MAB5492; 1:500 in 1% BSA in PBS-T) in mouse brain sections, the PolyStain TS kit (Biotrend) was used according to the manufacturer's instructions.

### Human

Sections were cut using microtome from fixed-frozen human tissue blocks in OCT compound and were used in a free floating immunohistochemical staining procedure. The sections were cut at either 30 – 50 μm and collected in 1 % sodium azide in PBS. Primary antibody: MID1 (2), 1:100 in normal goat serum; Secondary antibody: goat anti-rabbit biotin, Chemicon AP132B, 1:1,000 in normal goat serum; Tertiary antibody: streptavidin-HRP, Southern Technology 7100-05, 1:1,000 in normal goat serum. The sections were incubated with DAB solution (Sigma FAST Tablets D4293) and were mounted on a gelatine-coated glass slide.

Dried tissue sections were washed thoroughly in dH_2_O and incubated in staining solution for 15 to 20 min. Then, slides were quickly washed in dH_2_O and dehydrated in increasing amounts of ethanol for 5 min each (75 %, 85 %, 95 %), 2 times in 100 % ethanol for 10 min and 2 times in xylene for 10 min. Slides were covered with PERTEX and a coverslip.

## Quantitative real-time PCR

Total RNA was isolated using the RNeasy Plus Mini Kit (Qiagen) with QIAshredder colums (Qiagen). Next, cDNA synthesis reactions were prepared using the TaqMan reverse transcription reagents kit (Applied Biosystems), according to the manufacturer’s instructions (Roche) and the following temperature profile was used: 25 °C for 10 min, 48 °C for 1 h, 95 °C for 5 min, and cooling down to 4 °C. Finally quantitative real-time PCR was carried out using the SYBRGreen PCR master mix (Applied Biosystems, Nr. 4309155). Samples were analyzed in quadruplicates. Primers used were:

Name Sequence

hMID1_f CTGCCAGGTCTGGTGTCATG

hMID1_r AATCAGGCTTAGGGCCCTTCT

hRPL22_f TGACATCCGAGGTGCCTTTC

hRPL22_r GTTAGCAACTACGCGCAACC

mGAPDH_f GCACAGTCAAGGCCGAGAAT

mGAPDH_r GCCTTCTCCATGGTGGTGAA

mMID1_f CAAAGTGGCACCAAGTATATCTTCA

mMID1_r TCCGGGCTCGCTGCTA

## Quantitative image analysis

For quantification of MID1 IHC staining in human cortical sections the Definiens Developer XD 2.3

was used. The white matter as ROI was selected manually, the thresholds for signal detection were adjusted (0.35 for cresyl violet and 0.4 for DAB staining), and nucleus size was set to 40 µm.

# Supplementary Figures and Tables

## Supplementary Tables

Table 1: Clinical features of brain tissue donors (M, male; F, female; PMD, post-mortal delay)

| ID | Sex | Age | CAG repeat | PMD | qPCR | IHC |
| --- | --- | --- | --- | --- | --- | --- |
| C1 | M | 42 | -- | 14 |  | + |
| C2 | F | 64 | 18 / 23 | 6 | + | + |
| C3 | F | 59 | 15 / 17 | 21 | + | + |
| C4 | M | 41 | -- | 16 | + | + |
| C5 | M | 64 | 17 / 18 | 7 | + | + |
| C6 | M | 89 | -- | 19 | + | + |
| C7 | M | 48 | -- | -- | + |  |
| C8 | F | 78 | -- | -- | + |  |
| C9 | F | 89 | -- | -- | + |  |
| HD1 | M | 41 | 19 / 39 | 11 | + | + |
| HD2 | M | 40 | 18 / 51 | 15 | + | + |
| HD3 | F | 67 | 15 / 42 | 9 | + | + |
| HD4 | M | 75 | 19 / 43 | 3 | + | + |
| HD5 | F | 53 | 21 / 47 | 12 | + | + |
| HD6 | F | 57 | -- | -- | + |  |
| HD7 | M | 62 | -- | -- | + |  |
| HD8 | M | 48 | -- | -- | + |  |
| HD9 | M | 57 | 17/43 | -- | + | + |

1. Waldvogel HJ, Bullock JY, Synek BJ, Curtis MA, van Roon-Mom WM, Faull RL. The collection and processing of human brain tissue for research. Cell Tissue Bank. 2008;9(3):169-79.

2. Schweiger S, Matthes F, Posey K, Kickstein E, Weber S, Hettich MM, et al. Resveratrol induces dephosphorylation of Tau by interfering with the MID1-PP2A complex. Sci Rep. 2017;7(1):13753.
